# Potential Prognostic Biomarkers of Lung Adenocarcinoma Based on Bioinformatic Analysis

**DOI:** 10.1155/2021/8859996

**Published:** 2021-01-14

**Authors:** Jili Hou, Cheng Yao

**Affiliations:** ^1^Department of Medical Oncology, Zhuji People's Hospital of Zhejiang Province, The Zhuji Affiliated Hospital of Shaoxing University, No. 9 Jianmin Road, Tao Zhu Street, Zhuji, Zhejiang 311800, China; ^2^Department of Medical Oncology, The Second Affiliated Hospital of Zhejiang Chinese Medical University, 318 Chaowang Road, Hangzhou, Zhejiang 310005, China

## Abstract

Lung adenocarcinoma (LUAD), which accounts for 60% of non-small-cell lung cancers, is poorly diagnosed and has a low average 5-year survival rate (approximately 20%). It remains the leading cause of cancer-related deaths worldwide. Studies on long noncoding RNAs (lncRNAs) in LUAD-related competing endogenous RNA (ceRNA) networks are limited. We aimed to identify novel prognostic biomarkers for LUAD using bioinformatic tools and data analysis. We systemically integrated differentially expressed genes and clinically significant modules using weighted correlation network analysis. We performed a functional analysis of the collected candidate genes and explored three LUAD-related genes (*VWF*, *PECAM1*, and *COL1A1*) associated with the overall survival rates of patients with LUAD. Based on Cox proportional hazards analysis of candidate mRNAs and lncRNAs together with differentially expressed microRNAs, we constructed ceRNA networks, obtained 12 lncRNAs in the ceRNA networks, and revealed seven novel lncRNAs *AC021016.2*, *AC079630.1*, *AC116407.1*, *AC125807.2*, *AF131215.5*, *LINC01936*, and *RHOXF1-AS1*. These lncRNAs were found to be associated with overall survival rates and are suitable for the prediction of prognosis by Kaplan-Meier survival and receiver operating characteristic curve analyses. In particular, three lncRNAs—*AF131215.5*, *AC125807.2*, and *LINC01936*—showed an independent prognostic value of overall survival for patients with LUAD. We evaluated the diagnostic capabilities of seven lncRNAs for patients with LUAD using principal component analysis and the Gene Set Variation Analysis index. lncRNAs and crucial genes could be effectively used for distinguishing LUAD tumors from normal tissues in the Gene Expression Omnibus profile. In particular, *AC021016.2* showed a significant prognostic value in the validation dataset. Our findings reveal the significance of exploring lncRNAs in cancer-related ceRNAs using bioinformatic strategies.

## 1. Introduction

Lung cancer is the most commonly diagnosed cancer and a dominant cause of cancer-related deaths globally [1]. The high mortality rate relates to an overall 5-year survival rate estimated at 15% and unsatisfactory late diagnosis. Lung cancer can be classified into non-small-cell cancer (NSCLC, 85%) and small-cell lung cancer (SCLC, 15%) [2]. NSCLC includes three subgroups: lung squamous carcinoma (LUSC), lung adenocarcinoma (LUAD), and large-cell carcinoma subtypes [3]. Among the three subtypes, LUAD constitutes approximately 60% of NSCLCs and is the most frequently diagnosed and lethal subtype [4]. Due to the high mutational burden and complex tumor microenvironment, there is an urgent need to improve the diagnosis and therapies of LUAD and identify novel prognostic biomarkers and therapeutic targets for LUAD.

Long noncoding RNAs (lncRNAs) are noncoding RNAs with lengths greater than 200 bp. lncRNAs have been confirmed to be widely expressed in human cells and play essential roles in various biological processes and the progression of cancers such as lung cancer [5]. Previous research has shown that lncRNA SBF2-AS1 is vital for the tumorigenesis of early-stage LUAD [6]. lncRNA LINC00857 can predict poor survival of lung cancer patients and promote tumor progression via cell cycle regulation [7]. lncRNA HOXA11-AS promotes cisplatin resistance of human LUAD cells via the microRNA- (*miR-*)*454-3p*/Stat3 axis [8]. HCP5 is a SMAD3-responsive lncRNA that promotes LUAD metastasis via the *miR-203*/SNAI axis [9]. Therefore, identification of lncRNAs associated with LUAD may be of great value for exploring the occurrence and development of LUAD as well as for its diagnosis and evaluation.

Weighted correlation network analysis (WGCNA) is a systemic integration of R functions relying on weighted gene coexpression network analysis, a system biology method for exploring correlation patterns in microarray samples [10]. It was developed to identify highly correlated gene clusters (modules), summarize clusters based on the module eigengene or an intramodular hub gene, map modules to each other and the clinical signatures of samples, and calculate module membership measures. WGCNA has been employed to obtain the most significant modules of lncRNA, miRNA, and mRNA in LUSC and breast cancer progression [11, 12]. Correlation network analysis has been widely applied to identify candidate biomarkers or therapeutic targets in cancers. Hu et al. recently analyzed data related to LUSC in The Cancer Genome Atlas (TCGA), identified a three-lncRNA signature, and evaluated its potential value as a prognostic biomarker [11].

In this study, we aimed to identify novel prognostic biomarkers for LUAD using bioinformatic tools and data analysis.

## 2. Materials and Methods

### 2.1. Collection and Integration of Data from TCGA

To explore the differences in gene expression patterns between primary tumor and solid tissue (normal), we queried the TCGA database and selected the TCGA-LUAD project for our study. The duplicated samples (two samples in RNA sequencing (RNA-seq) and miRNA-seq) were removed; primary tumor and solid tissue (normal) samples were reserved for downstream analysis; 20 samples with the nonprimary tumors or normal solid tissue in RNA-seq and six samples in miRNA-seq were deleted. Finally, RNA-seq of 572 samples (513 primary tumors and 59 normal solid tissue) and miRNA-seq of 559 samples (513 primary tumors and 46 normal solid tissue) were merged and integrated for further analysis. The corresponding clinical data were collected and integrated from TCGA.

### 2.2. Data Preprocessing and Identification of DERNAs

All RNA-seq and miRNA-seq data in the TCGA-LUAD project were normalized using the quantile method in the limma package [13]. Genes with low expression levels were removed. The differentially expressed RNAs (DERNAs) were screened using the edgeR package in the Bioconductor project (http://www.bioconductor.org/). ∣log_2_(fold change (FC)) | >1.5 and false discovery rate < 0.05 were set as the cut-off criteria. The ggplot2 package (https://github.com/tidyverse/ggplot2) was used to construct the volcano plots of differentially expressed genes (DEGs) and DEmiRNAs. Pheatmap software was applied to visualize DERNAs using a heatmap.

### 2.3. Identifying Crucial Modules That Significantly Map to LUAD

WGCNA was performed to construct the gene coexpression network of genes and to identify coexpression gene modules. We employed soft-threshold power *β* = 7 and minimum module size = 180 to determine coexpression gene modules and classify clinically significant modules.

### 2.4. Protein-Protein Interaction (PPI) and Functional Enrichment Analysis of Candidate Genes

The genes overlapping the identified DERNAs and the most prominent module in WGCNA were considered candidate genes for further analysis. PPI and functional enrichment analyses of candidate genes were performed using the DAVID database (https://david.ncifcrf.gov/) for the annotation, visualization, and integrated discovery of genes. Gene Ontology (GO) and Kyoto Encyclopedia of Genes and Genomes (KEGG) enrichment analyses were carried out using the DAVID database and visualized in a bubble plot using the R package. The PPI was analyzed using STRING (https://string-db.org/), an online tool providing functional protein association networks. The outputs obtained from GO analysis and PPI interaction network were visualized using Cytoscape software (https://cytoscape.org/). Crucial genes were predicted using cytoHubba (http://apps.cytoscape.org/apps/cytohubba), a Cytoscape plugin.

### 2.5. Construction of ceRNA Networks Based on Candidate DEGs

Genes overlapping with DEGs and the most significant module in WGCNA with prognostic value verified by univariate Cox proportional hazards regression analysis (*P* < 0.05) were collected to construct the lncRNA-miRNA-mRNA ceRNA network. Database miRcode (http://www.mircode.org/) and miRTarBase version 7.0 (http://mirtarbase.mbc.nctu.edu.tw/) were utilized to predict lncRNA-miRNA and miRNA-mRNA interactions. The Pearson correlation coefficient was used to identify the expression correlation among lncRNAs, miRNAs, and mRNAs in ceRNA pairs. The regulation similarity of miRNAs in the regulation of lncRNAs and mRNAs was considered. Subsequently, the ceRNA network was constructed and visualized using Cytoscape.

### 2.6. Crucial DEG Classification and Gene Set Variation Analysis (GSVA) Index Construction

To assess gene set enrichment among samples, GSVA was performed using a nonparametric method. The features of candidate DEGs were scored and calculated in samples using GSVA [14]. Then, a matrix containing the candidate genes' GSVA index was obtained. To evaluate the diagnostic value of candidate DEGs, receiver operating characteristic (ROC) analysis was conducted based on the GSVA score. We scanned the candidate lncRNAs, evaluated their relationship to clinical traits (such as survival status), and identified critical lncRNAs based on the Kaplan-Meier (KM) method. Multivariate Cox regression analysis was performed to explore how candidate lncRNAs jointly influence the survival of patients with LUAD. To assess whether the gene matrix could distinguish LUAD from healthy samples, principal component analysis (PCA) was conducted using the FactoMineR function and visualized via the factoextra package (https://CRAN.R-project.org/package=factoextra).

### 2.7. Evaluating Candidate Genes with Data from the GEO Dataset

Gene expression profiles from a recent LUAD-related study on Chinese people were downloaded to evaluate the prognostic significance of candidate genes and their potential as biomarkers for LUAD [15]. Gene expression and clinical data were collected and integrated from Gene Expression Omnibus (GEO; GSE140343) and supplementary profiles.

## 3. Results

### 3.1. DERNA Identification Related to LUAD

**Figure 1:**
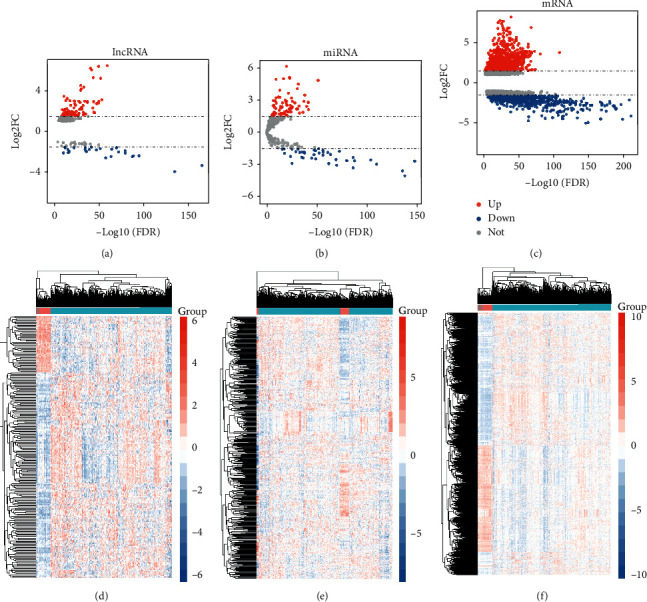
Differentially expressed (DE)RNAs in lung adenocarcinoma (LUAD). (a–c) Volcano plot of DE long noncoding RNAs (lncRNAs) (a), microRNAs (miRNAs) (b), and mRNAs (c). The *x*-axis represents the log-transformed false discovery rate (FDR), and the *y*-axis represents the mean expression differences. (d–f) Heatmap of DElncRNAs (d), DEmiRNAs (e), and DEmRNAs (f) between LUAD and normal lung tissue samples. The *x*-axis represents DERNAs, and the *y*-axis represents the samples.

We utilized a total of 60,483 genes from 572 samples in RNA-seq and 2,588 miRNAs from 559 samples in miRNA-seq for DERNA analysis. We obtained 1,861 DERNAs including 1,615 mRNAs (974 upregulated and 641 downregulated) and 108 lncRNAs (81 upregulated and 27 downregulated) and visualized the distribution of FC and *P* value in a volcano plot (Figures 1(a) and 1(c)). We observed 114 differentially regulated miRNAs (74 upregulated and 40 downregulated) and depicted the distribution with a volcano plot (Figure 1(b)). The expression level of DERNAs was visualized using a heatmap (Figures 1(d)–1(f)).

### 3.2. The Analysis of LUAD-Related Significant Modules Using WGCNA

**Figure 2:**
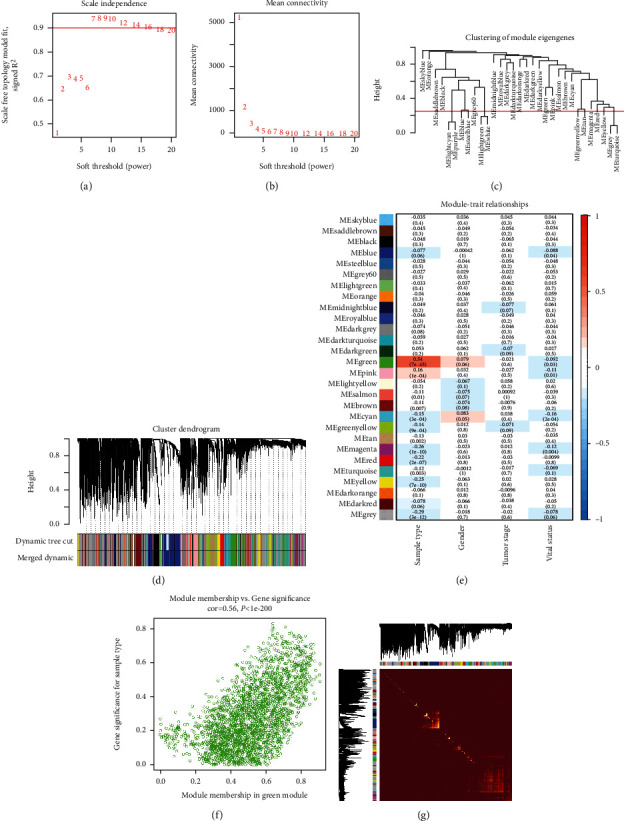
Weighted gene coexpression network analysis (WGCNA) of lung adenocarcinoma (LUAD). (a) Network topology of various soft-threshold power analysis. (b) The property test of the scale-free network. (c) Hierarchical clustering dendrogram of module eigengenes. (d) Cluster dendrogram of the coexpression network modules based on topological overlapping differentially expressed genes (DEGs). (e) Identification of the most significant LUAD-related module (sample type refers to the primary tumor or solid tissue). (f) Scatterplot of gene significance (*y*-axis) versus module membership (*x*-axis) in the most significant module (green module). (g) Heatmap plot of topological overlap in the gene network.

Gene expression (RNA-seq) and clinical data were employed to construct the coexpression network using the WGCNA algorithm. The Pearson correlation matrix of genes was converted into a strengthened adjacency matrix by soft-threshold power *β* = 7 based on the scale-free topology criterion with *R*^2^ = 0.9 (Figures 2(a) and 2(b)). Thirty-one module eigengenes were clustered (Figure 2(c)), merged, and integrated. The coexpression network modules (31 initial modules and 27 merged modules) based on topological overlapping DEGs were identified (Figure 2(d)). The heatmap revealed the correlation network of the modules (Figure 2(g)). The correlation analysis between the modules and clinical traits indicated that the green module (cor = 0.54 and *P* = 7*e* − 45, containing 2,457 genes) was most significantly associated with LUAD compared with the other 26 modules (Figure 2(e)). The module membership in the green module possessed the most significant correlation (cor = 0.56 and *P* < 1*e* − 200; Figure 2(f)).

### 3.3. Functional Enrichment Analysis of Candidate Genes and PPI Network Construction

**Figure 3:**
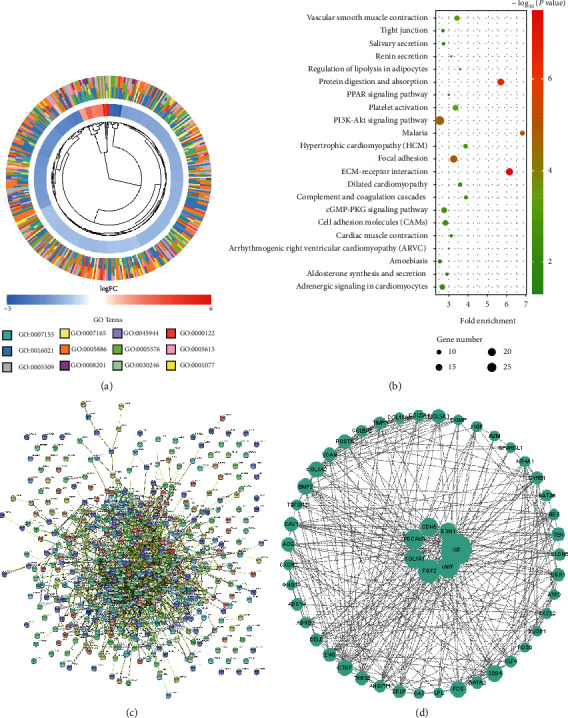
Function enrichment analysis and protein-protein interaction (PPI) of candidate differentially expressed genes (DEGs). (a) Cluster plot of Gene Ontology (GO) terms; the fold change of genes is depicted in color (blue: low expression, red: high expression); the GO terms are visualized in different colors. (b) Bubble plot of Kyoto Encyclopedia of Genes and Genomes (KEGG) pathways; the *x*-axis indicates fold enrichment level; the *y*-axis shows terms of pathways; the bubble size indicates the number of genes in the pathway; the significance level (*P* value) was ranked and colorized. (c) The PPI of DEGs obtained from the STRING database. (d) Top 50 hub DEGs were collected and visualized using Cytoscape (the size of circles represents the scores of “degree”).

We overlapped and integrated 2,457 genes in the green module and 1,861 DEGs; 529 DEGs—candidate genes—were obtained (including 501 DEmRNAs) for further analysis. GO enrichment analysis indicated that these candidate genes were enriched in 273 GO terms, including 198 for biological process, 34 for cellular component, and 41 for molecular function. The top four of each category were visualized, such as the plasma membrane, extracellular space, and regulation of transcription from RNA polymerase II promoter (Figure 3(a)). KEGG enrichment analysis showed that the PI3K-AKT signaling pathway contained the majority of the DEGs (Figure 3(b)), which are involved in many cellular functions such as proliferation and survival. A total of 501 differentially expressed mRNAs were used for the PPI network construction using the STRING database (Figure 3(c)). The top 50 DEmRNAs were collected and visualized using Cytoscape (Figure 3(d)).

### 3.4. Survival and ROC Curve Analysis

**Figure 4:**
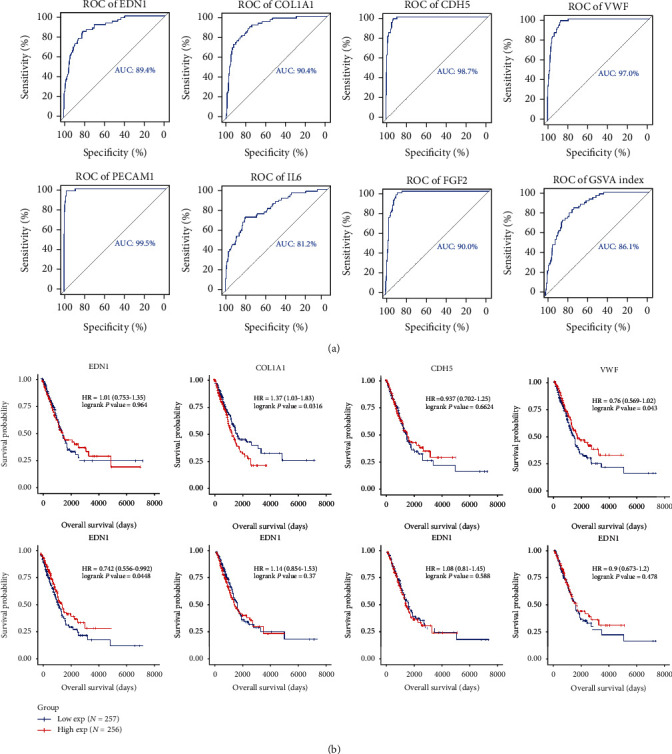
(a) Receiver operating characteristic (ROC) curve of top seven crucial genes; (b) Kaplan-Meier plot of seven critical genes.

Seven genes (*EDN1*, *COL1A1*, *CDH5*, von Willebrand factor (*VWF*), *PECAM1*, *IL6*, and *FGF2*) were identified with the degree score > 40 and viewed as crucial genes related to LUAD. ROC curve analysis demonstrated that the area under the curve (AUC) values of seven genes exceeded 0.8. The ROC curve of the GSVA index based on the seven crucial genes showed that the AUC score was 86.1%, indicating good performance in distinguishing patients with LUAD from healthy individuals (Figure 4(a)). However, no significant association was identified among the seven critical gene GSVA score matrix and the overall prognosis of patients using KM plot analysis (*P* = 0.478; Figure 4(b)). Three genes (*VWF*, *PECAM1*, and *COL1A1*) were found to correlate with overall survival (OS) in patients with LUAD and distinguish them from healthy individuals (AUC score > 0.9).

### 3.5. mRNA-miRNA-lncRNA ceRNA Network Construction Related to LUAD

**Figure 5:**
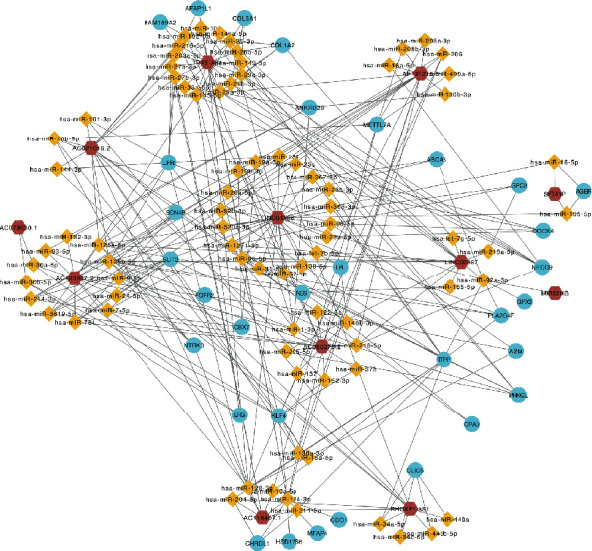
LUAD-related long noncoding RNA- (lncRNA-) microRNA- (miRNA-) mRNA competing endogenous RNA (ceRNA) network. The ceRNA network contained 12 lncRNAs (red hexagons), 79 miRNAs (yellow diamonds), and 32 mRNAs (blue circles).

The ceRNA network was constructed based on LUAD-related 13 DElncRNAs, 168 DEmRNAs (which were collected by overlapping DEG analysis and WGCNA and showed a significant prognostic value in univariate Cox regression analysis with *P* < 0.05), and DEmiRNAs. We obtained a ceRNA network consisting of 12 lncRNAs, 79 miRNAs, and 32 mRNAs (Figure 5). The 12 lncRNAs that formed a network related to LUAD were collected for further analysis.

### 3.6. ROC and KM Analysis of 12 lncRNAs in ceRNA Networks Related to LUAD

**Figure 6:**
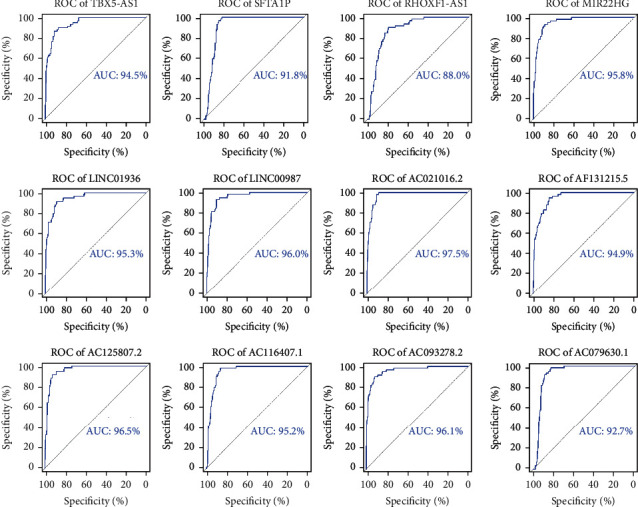
Receiver operating characteristic (ROC) curves of 12 candidate long noncoding RNAs (lncRNAs). The area under the curve (AUC) under the binomial exact confidence interval was calculated to generate the ROC curve.

**Figure 7:**
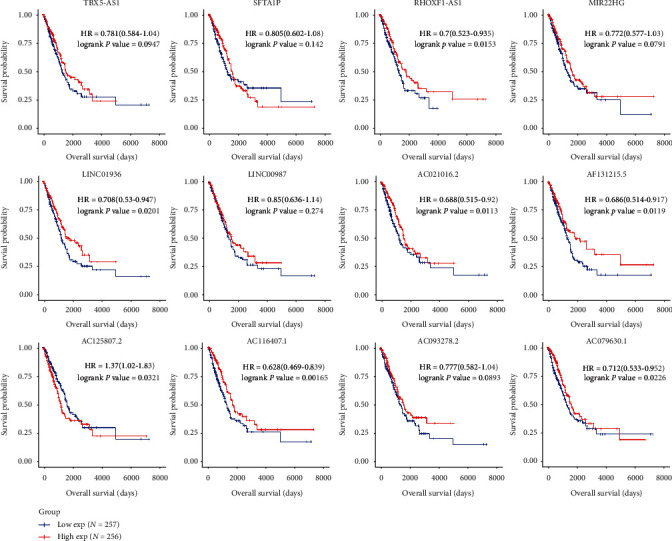
Kaplan-Meier survival of 12 candidate long noncoding RNAs (lncRNAs). Corresponding Kaplan-Meier plots of 12 lncRNAs are depicted; the red line refers to high expression, and blue refers to low expression.

ROC and KM survival curves were generated to illustrate the connection between the 12 lncRNAs and LUAD prognosis. All 12 lncRNAs displayed sound performance in distinguishing LUAD patients from healthy controls (Figure 6). The lowest AUC score (*RHOXF1-AS1*) reached 88.0%, which was also the only one that was below 90%, and that of AC021016.2 reached 97.5%. Seven lncRNAs—*AC021016.2*, *AC079630.1*, *AC116407.1*, *AC125807.2*, *AF131215.5*, *LINC01936*, and *RHOXF1-AS1*—showed potential prognostic value with *P* < 0.05. These results show that patients with a higher expression of *AC125807.2* (Figure 7) had a lower probability of survival than those with a lower expression of *AC125807.2*. Higher expression levels of six genes, including *AC021016.2*, *AC079630.1*, *AC116407.1*, *AF131215.5*, *LINC01936*, and *RHOXF1-AS1*, in patients with LUAD showed better survival prognoses (Figure 7).

### 3.7. Evaluation of Seven lncRNAs as Biomarkers of LUAD

**Figure 8:**
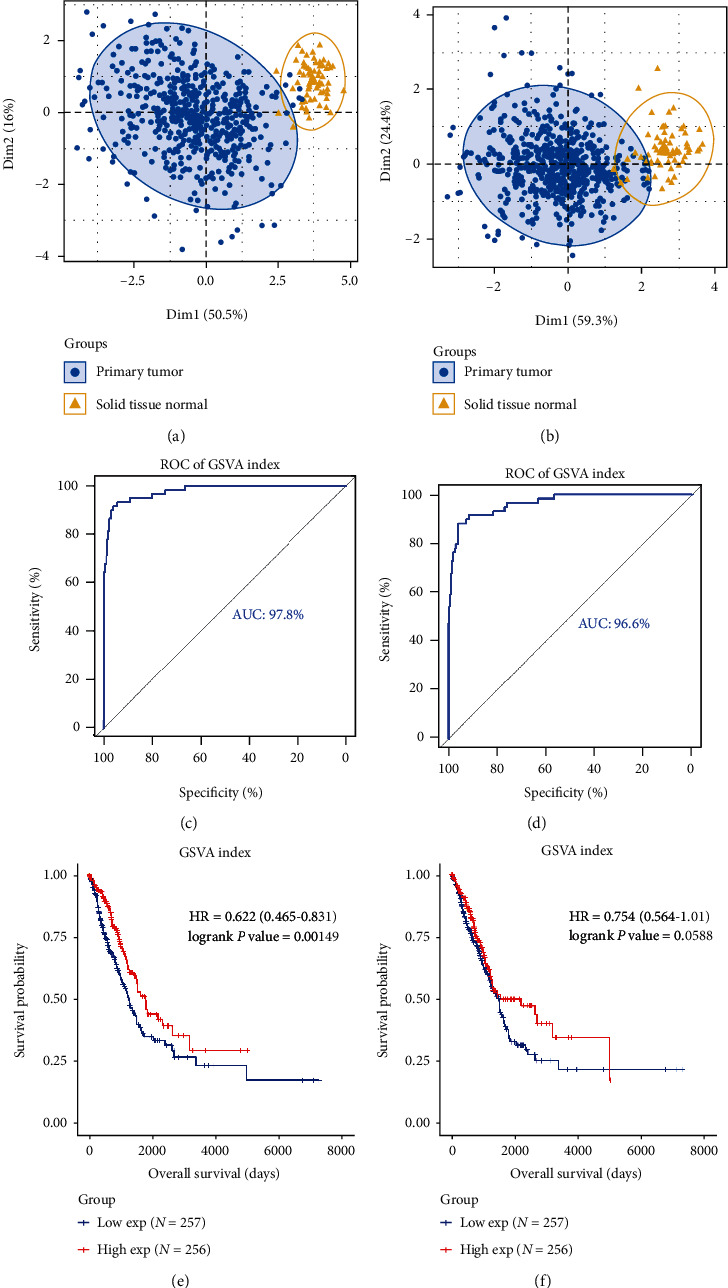
Efficacy of seven candidate long noncoding RNAs (lncRNAs) as biomarkers. (a, b) Principal component analysis (PCA) plot is based on the expression of seven candidate lncRNAs and three lncRNAs with independent prognostic values. The receiver operating characteristic (ROC) curve of the Gene Set Variation Analysis (GSVA) index of candidate lncRNAs (c, d) and Kaplan-Meier survival plot of the GSVA index (e, f) is depicted.

We obtained seven candidate lncRNAs based on the ceRNA network, ROC, and KM survival analyses. The PCA plot demonstrated that the expression of these lncRNAs could effectively distinguish patients with LUAD from healthy individuals (Figure 8(a)). Compared with seven lncRNAs, the GSVA index based on seven lncRNAs showed higher discriminatory ability with an AUC of the ROC curve of 0.978 (Figure 8(c)). The matrix also showed good performance in terms of prognosis (Figure 8(e)). Seven candidate lncRNAs were merged with a linear model for multivariate Cox regression analysis. We observed that all three overall tests (likelihood ratio, Wald, and score) indicated significance of the model with *P* value < 0.05. Covariates *AF131215.5*, *AC125807.2*, and *LINC01936* remained significant. However, covariates *AC021016.2* (*P* = 0.99), *AC079630.1* (*P* = 0.26), *AC116407.1* (*P* = 0.19), and *RHOXF1-AS1* (*P* = 0.53) were not significant after adjusting for other lncRNAs (Table 1). The boundary between tumor and normal tissues was clearer in PCA with seven lncRNAs (Figure 8(a)) than in those with three lncRNAs (*AF131215.5*, *AC125807.2*, and *LINC01936*) with independent prognostic values (Figure 8(b)). Compared to GSVA without the four lncRNAs (without independent prognostic values), GSVA containing all seven lncRNAs was more powerful in discriminating tumor tissues (AUC of 0.978 for seven lncRNAs and AUC of 0.966 for three independent lncRNAs) (Figures 8(c) and 8(d)), whereas GSVA established using the three lncRNAs showed no prognostic significance (Figure 8(f)).

### 3.8. Validation of Candidate Genes with GEO Dataset

**Figure 9:**
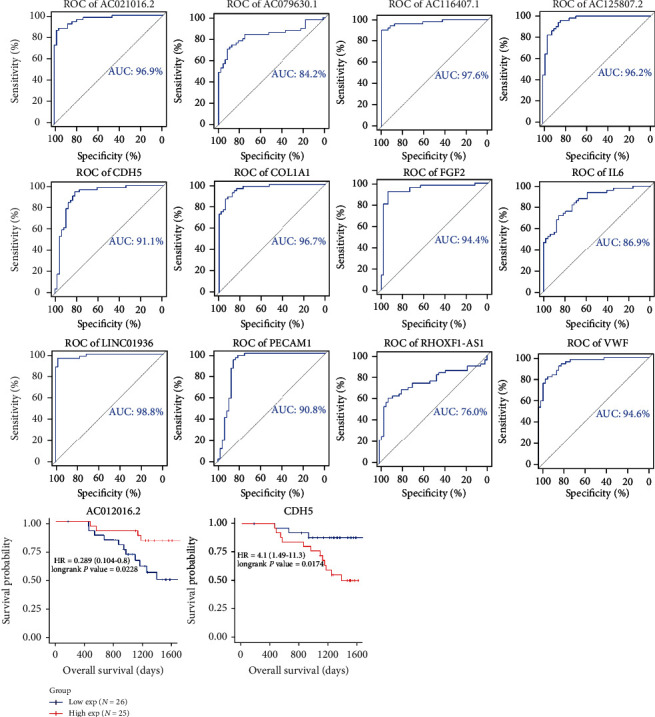
Validation of candidate genes with Gene Expression Omnibus (GEO; GSE140343). The receiver operating characteristic (ROC) curve and Kaplan-Meier plot were used to evaluate the candidate genes' prognostic significance and potential as biomarkers for identifying patients with lung adenocarcinoma (LUAD).

The expression of 100 samples (51 LUAD tumors and 49 clinical normal lung tissues) was assessed for the validation process of seven crucial genes from PPI and seven critical lncRNAs. The examination of *AF131215.5* and *EDN1* failed due to the absence of expression information, which was dropped by the submitters. Six identified crucial genes *IL6* (with the lowest AUC = 0.869), *FGF2*, *VWF*, *PECAM1*, *COL1A1*, and *CDH5* showed good performance in identifying LUAD tumors. lncRNAs showed the potential to distinguish LUAD tumors from normal tissues (Figure 9). KM analysis showed that two genes *AC021016.2* (*P* = 0.023) and *CDH5* (*P* = 0.018) showed prognostic significance in Chinese patients with LUAD (Figure 9).

## 4. Discussion

We obtained 1,615 DEmRNAs (Figures 1(c) and 1(f)) and 108 lncRNAs (Figures 1(a) and 1(d)) along with 114 differentially regulated miRNAs (Figures 1(b) and 1(e)) from DE analysis of TCGA-LUAD. We observed 31 modules based on WGCNA of RNA-seq of the LUAD project with soft power = 7 and minimum module = 180 and merged them into 27 modules with a correlation of 0.75 (Figures 2(c) and 2(d)). The module “green” containing 2,457 genes showed that it is highly correlated with LUAD, correlation = 0.56 and *P* < 1*e* − 200 (Figures 2(e) and 2(f)). We collected, integrated, and overlapped DEGs (mRNAs and lncRNAs) and genes in module “green” and studied 501 mRNAs and 22 lncRNAs. We detected seven crucial genes—*IL6*, *FGF2*, *VWF*, *PECAM1*, *COL1A1*, *CDH5*, and *EDN1*—based on the PPI analysis of 501 mRNAs. The GSVA index of seven critical genes showed superior performance in distinguishing patients with LUAD from healthy subjects. Three genes (*VWF*, *PECAM1*, and *COL1A1*) were associated with OS in patients with LUAD and showed superior performance in distinguishing patients with LUAD from healthy individuals (AUC score > 0.9). *VMF* is upregulated by GATA3 in the LUAD vasculature [16]. Serum VWF can be employed for the early diagnosis of LUAD in patients with type 2 diabetes mellitus [17]. The imbalance between VWF secretion and ADAMTS-13 plays a critical role in the hypercoagulability state in advanced NSCLC [18]. *PECAM1* was reported to be involved in lung repair and regeneration in acute respiratory distress syndrome [19] whereas *COL1A1* is correlated with hypoxia markers in NSCLC [20]. The *miR-196a*/COL1A1 axis is known to be regulated by lncRNA H19 in pulmonary fibrosis [21].

The GO functional enrichment analysis of the candidate genes overlapping DEGs and WGCNA showed that the candidate genes were enriched in cellular component terms such as plasma membrane and extracellular space, which is consistent with previous research [22]. These candidate genes were also significantly enriched in the regulation of transcription from RNA polymerase II promoter, which is in line with a study on HOXA13 in LUAD [23].

We constructed ceRNA networks based on 13 DElncRNAs, 168 DEmRNAs with prognostic value (*P* < 0.05 in univariate Cox regression analysis), and DEmiRNAs. As a result, 12 lncRNAs, 79 miRNAs, and 32 mRNAs were identified in the ceRNA network (Figure 5). Numerous researchers have reported the relationship between lncRNAs in ceRNAs and LUAD [24, 25]. We identified seven lncRNAs—*AC021016.2*, *AC079630.1*, *AC116407.1*, *AC125807.2*, *AF131215.5*, *LINC01936*, and *RHOXF1-AS1*—associated with the OS rates of patients with LUAD by integrating DEG analysis, WGCNA, univariate Cox regression, KM analysis, and ceRNA construction. Three lncRNAs (*AF131215.5*, *P* = 0.047; *AC125807.2* with *P* = 0.018; and *LINC01936* with *P* = 0.011) were significant in multivariate Cox regression analysis (Table 1). Increased expression of *AF131215.5* and *LINC01936* showed a strong relation to the decreased risk of death, with a hazard ratio (HR) of 0.88 and 0.86, respectively. However, *AC125807.2* showed a reverse correlation with HR = 1.19. By contrast, the significant values of *AC021016.2*, *AC079630.1*, *AC116407.1*, and *RHOXF1-AS1* were larger than the threshold (0.05), and their confidence interval was 1, which indicates that the expression of these four genes contributed little to the change in HR after adjusting for others. We identified that *miR-125b* targeted by five OS-related lncRNAs (*AC125807.2*, *AC021016.2*, *LINC01936*, *AF131215.5*, and *RHOXF1-AS1*) could regulate the expression of the five mRNAs—*NTRK3*, *FGFR2*, *LIFR*, *PLA2G4F*, and *NES* (one of top 50 critical genes). Biamonte et al. demonstrated that the downregulation of *miR-125b* stimulates the apoptosis of NSCLC cells by enhancing the expression of the p53 protein [26]. Niu et al. reported the potential prognostic value of *NTRK3* mutation in patients with LUAD treated with immune checkpoint inhibitors [27]. The membrane receptor FGFR2 drives LUAD progression through aberrant protein-protein interactions mediated via its C-terminal proline-rich motif [28]. *miR-124-3p*, targeted by two OS-LUAD-related lncRNAs (AC116407.1 and AF131215.5), was predicted to mediate the expression of the seven genes (*AFAP1L1*, *CDO1*, *MFAP4*, *METTL7A*, *CHRDL1*, *HSD17B6*, and *KLF4*). Several researchers have demonstrated the role of *miR-124-3p* in the regulation of multiple cancers such as bladder and pancreatic cancers and hepatocellular carcinoma [29–31]. Previous research revealed that OGFRP1 promoted the progression of NSCLC, partly due to the upregulation of LYPD3 expression by sponging *miR-124-3p* [32]. *AFAP1L1* has been reported to mediate proliferation and survival in NSCLC [33]. *CDO1* is a metabolic liability for NSCLC [34], and the functional identification of cancer-specific methylation of CDO1 could be applied for the diagnosis of lung cancer [35]. The expression of MFAP4 is negatively regulated by *miR-147b* in LUAD cells [36]. The deubiquitinase USP10 moderates KLF4 stability and suppresses lung tumorigenesis [37]. Although researchers have reported on some miRNAs and genes in ceRNA networks, further experimental methods are still required to reveal the efficacy and mechanisms of these ceRNA networks in regulating LUAD.

We further validated candidate genes from TCGA with expression profiles related to Chinese patients with LUAD. Despite the absence of the expression of the two genes, both lncRNAs and protein-coding genes showed good performance in distinguishing LUAD tumors from normal tissues (Figure 9). The AUC scores of four lncRNAs and five mRNAs were greater than 0.9. However, only two genes, *AC021016.2* (*P* = 0.023) and *CDH5* (*P* = 0.018), showed prognostic significance (Figure 9). The decreased probability of the OS of patients with LUAD in GEO is consistent with the findings observed in TCGA-LUAD data. However, we observed no overlapping mRNAs or lncRNAs with a previous study that did similar research on LUAD by constructing a ceRNA network with DEGs using Cytoscape [38]. This might be caused by the following reasons: the definitions set by the two studies were different (we set ∣log_2_FC | >1.5 instead of ∣log_2_FC | >2); we added two processes before ceRNA network construction, including collecting LUAD-related genes (with differential expression) based on WGCNA and evaluating the prognostic value of genes with univariate Cox regression; when constructing the ceRNA network, besides interactions predicted by the database, two rules (the correlation between the expression of lncRNAs and mRNAs and similar roles of miRNAs in regulating their corresponding lncRNAs and mRNAs) were considered. We examined the prognostic significance of seven reported lncRNAs in the study with Chinese LUAD data from GEO. Only one lncRNA, *H19*, was observed in the expression matrix (others were disregarded by the submitter due to low expression) but failed to be a significant prognostic marker with *P* = 0.576. These differences indicate the remarkable variability of molecular signatures of tumor tissues as markers for phenotypic traits, including the findings in this study (prognostic significance in this study). Owing to the complicated evolution of Darwinian-like processes of spontaneous tumors, individual tumors contain a unique clone with spatial and temporary heterogeneity [39]. We observed the stability of *AC021016.2* as a potential biomarker with prognostic significance. However, the genes showed an almost consistent ability to distinguish tumors from normal samples in the matrices of TCGA and GEO, and the genetic and nongenetic diversity of tumor samples contributes to phenotypic heterogeneity, as demonstrated by Marusyk et al. [39].

Apart from establishing a LUAD-related ceRNA network based on DEGs, we collected and integrated DEG outputs and clinical trait-related modules of WGCNA. Combined with univariate Cox proportional hazards and Pearson correlation analysis, we constructed ceRNA networks with potential clinical significance based on comprehensive strategies. Three lncRNAs (*AF131215.5*, *AC125807.2*, and *LINC01936*) indicated independent prognostic value; one lncRNA, *AC021016.2*, showed good performance in identifying patients with LUAD and prognostic value in TCGA in the GEO database. We utilized the GSVA index to systemically integrate the signatures of seven lncRNAs, correlated with OS rates predicted by KM survival analysis, to enhance its ability to identify LUAD-related patients and prognosis. We further compared the power of three independent lncRNAs and their joint impact on identifying tumor samples and prognostic significance (Figure 8). This indicated that four lncRNAs contributed to the recognition of tumor tissues, although not independently and prognostically significant.

## 5. Conclusions

We identified seven OS-associated lncRNAs in LUAD-related ceRNAs with good performance in distinguishing patients with LUAD in the TCGA database. Three lncRNAs *AF131215.5*, *AC125807.2*, and *LINC01936* represented the independent prognostic significance of OS in patients with LUAD in TCGA. The prognostic value of *AC021016.2* and its potential for distinguishing patients with LUAD from healthy subjects were confirmed by its profile from the GEO database.

## Figures and Tables

**Table 1:** Multivariate Cox regression analysis of lncRNAs. Hazard ratio (HR) and upper and lower 95% confidence intervals are listed in the table.

Symbol	coef	HR	Lower 95	Upper 95	*P* value
*AC021016.2*	-0.0009	0.9991	0.8119	1.2294	0.9932
*AC079630.1*	0.0503	1.0516	0.9635	1.1477	0.2599
*AC116407.1*	-0.0873	0.9164	0.8035	1.0453	0.1935
*AF131215.5*	-0.1331	0.8754	0.7679	0.9980	0.0466
*AC125807.2*	0.1779	1.1947	1.0307	1.3848	0.0182
*LINC01936*	-0.1544	0.8569	0.7612	0.9646	0.0106
*RHOXF1-AS1*	-0.0272	0.9732	0.8941	1.0594	0.5305

## Data Availability

The datasets used and/or analyzed during the current study are available from the corresponding author on reasonable request.
